# Paeonol Suppresses Chondrosarcoma Metastasis through Up-Regulation of miR-141 by Modulating PKCδ and c-Src Signaling Pathway

**DOI:** 10.3390/ijms150711760

**Published:** 2014-07-02

**Authors:** Chi-Ting Horng, Po-Chuen Shieh, Tzu-Wei Tan, Wei-Hung Yang, Chih-Hsin Tang

**Affiliations:** 1 Medical Education Center, Kaohsiung Armed Force General Hospital, Kaohsiung 802, Taiwan; E-Mail: mtc9042@yahoo.com.tw; 2 Department of Pharmacy, Tajen University, Pingtung 907, Taiwan; E-Mail: pochuen@mail.tajen.edu.tw; 3 Graduate Institute of Basic Medical Science, China Medical University, Taichung 404, Taiwan; E-Mail: twtan@mail.cmu.edu.tw; 4 Department of Pharmacology, School of Medicine, China Medical University, Taichung 404, Taiwan; 5 Department of Orthopedic Surgery, Taichung Hospital, Ministry of Health and Welfare, Taichung 403, Taiwan; E-Mail: u766018@ms42.hinet.net; 6 School of Chinese Medicine, China Medical University, Taichung 404, Taiwan; 7 Department of Nursing, National Taichung University of Science and Technology, Taichung 403, Taiwan; 8 Graduate Institute of Biotechnology, National Chung Hsing University, Taichung 402, Taiwan; 9 Department of Biotechnology, College of Health Science, Asia University, Taichung 413, Taiwan

**Keywords:** paeonol, chondrosarcoma, miR-141, metastasis

## Abstract

Chondrosarcoma, a primary malignant bone cancer, has potential for local invasion and distant metastasis, especially to the lungs. Patients diagnosed with it show poor prognosis. Paeonol (2'-hydroxy-4'-methoxyacetophenone), the main active compound of traditional Chinese remedy *Paeonia lactiflora* Pallas, exhibits anti-inflammatory and anti-tumor activity; whether paeonol regulates metastatic chondrosarcoma is largely unknown. Here, we find paeonol do not increase apoptosis. By contrast, at non-cytotoxic concentrations, paeonol suppresses migration and invasion of chondrosarcoma cells. We also demonstrate paeonol enhancing miR-141 expression and miR-141 inhibitor reversing paeonol-inhibited cell motility; paeonol also reduces protein kinase C (PKC)δ and c-Src kinase activity. Since paeonol inhibits migration and invasion of human chondrosarcoma via up-regulation of miR-141 via PKCδ and c-Src pathways, it thus might be a novel anti-metastasis agent for treatment of metastatic chondrosarcoma.

## 1. Introduction

Chondrosarcoma is derived from abnormal proliferation cartilage and accounts for about 26% of bone cancer. It usually occurs in males aged 10–80 years, most tumors appearing on the scapula, sternum, ribs, or pelvis [1,2]. In clinical practice, surgical resection remains the primary mode of therapy. Chondrosarcoma is reported as easily metastasizing to other organs: e.g., lung, liver [3,4]. Distant metastasis means poor prognosis and high incidence of fatality associated with this mesenchymal malignancy due to lack of an effective adjuvant therapy, making it important to explore novel remedies [5,6].

Tumor invasion and metastasis are prominent biological traits of cancer cells [7]. Mortality in such cases chiefly results from spread to distant organs. The microRNAs (miRNAs) are small (about 22-nucleotides long), non-coding RNAs that can modulate targeted gene expression through either translational repression or mRNA cleavage. One miRNA can regulate a set of functionally relevant genes simultaneously, which may reinforce phenotypic change [8,9], miRNAs control gene expression by binding to complementary 3'UTR sequences of target mRNAs [10,11]. Deregulated expression of miRNAs is reported in human cancer and may affect multiple steps during metastasis [12]. Recent studies report miRNAs involved in metastatic progression [13,14]. On the other hand, they are cited for mediating chondrosarcoma progression and metastasis [15,16], making miRNA a novel target for chondrosarcoma therapy.

Paeonol (2'-hydroxy-4'-methoxyacetophenone) is a key active compound of *Paeonia lactiflora.* Pallas is a traditional Chinese herb used in Asia and Europe to improve blood flow while suppressing expression of cyclooxygenase-2, nitric oxide synthase, as well as cell surface adhesion molecules TNF-α and IL-1β [17,18]. This compound has anti-oxidant and anti-inflammatory activity and demonstrably suppresses tumor formation [17,19,20]; it is also reported as reducing metastasis of human cancer [21,22], yet its effects on metastasis by human chondrosarcoma cells are largely unknown. Here, we report paeonol inhibiting migration and invasion of human chondrosarcoma cells. In addition, up-regulation of the miR-141 through protein kinase C (PKC)δ and c-Src pathways are involved in paeonol-reduced cell motility. Data point to paeonol as an anti-metastatic agent for treatment of metastasic chondrosarcoma.

## 2. Results

### 2.1. Paeonol Does not Induce Cell Death in Human Chondrosarcoma

**Figure 1:**
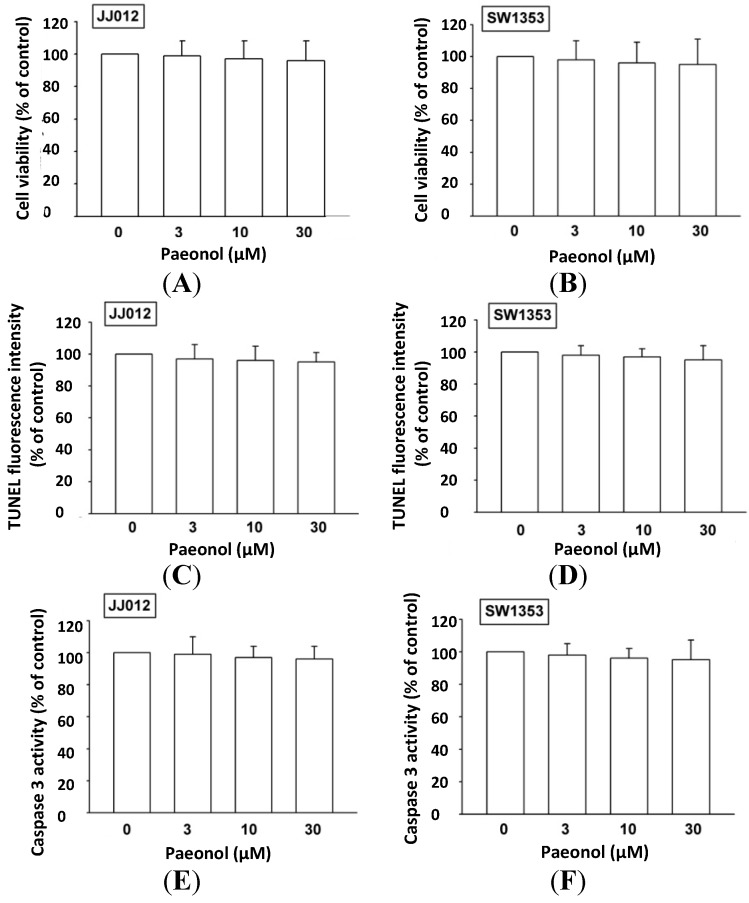
Paeonol does not induce apoptosis in human chondrosarcoma. (**A**,**B**) Cells were incubated with various concentrations of paeonol for 48 h, viability examined by 3-(4,5-dimethylthiazol-2-yl)-2,5-diphenyltetrazolium bromide (MTT) assay (*n* = 5); (**C**,**D**) Cells were incubated with paeonol for 48 h, triphosphate nick-end labeling (TUNEL)-positive ones were examined by flow cytometry (*n* = 6); and (**E**,**F**) Cells were incubated with paeonol for 24 h, and caspase 3 activity was examined by caspase 3 ELISA kit (*n* = 5). Results are expressed as mean ± S.E.M.

Paeonol has been reported to promote cancer apoptosis [17,19,20]; we investigated whether paeonol induced cell death in human chondrosarcoma. Cytotoxic effect was examined by 3-(4,5-dimethylthiazol-2-yl)-2,5-diphenyltetrazolium bromide (MTT) assay. Incubation of chondrosarcoma cells (JJ012 and SW1353) for 48 h did not affect viability (Figure 1A,B). We examined whether paeonol induced apoptosis in human chondrosarcoma cells by triphosphate nick-end labeling (TUNEL) staining and caspase 3 activity assay. Incubation of these cells with paeonol did not enhance TUNEL expression or caspase 3 activity (Figure 1C–F), indicating paeonol does not induce cell death in human chondrosarcoma. All subsequent experiments used this concentration range.

### 2.2. Paeonol Reduces Cell Migration, Wound-Healing Migration, and Cell Invasion

**Figure 2:**
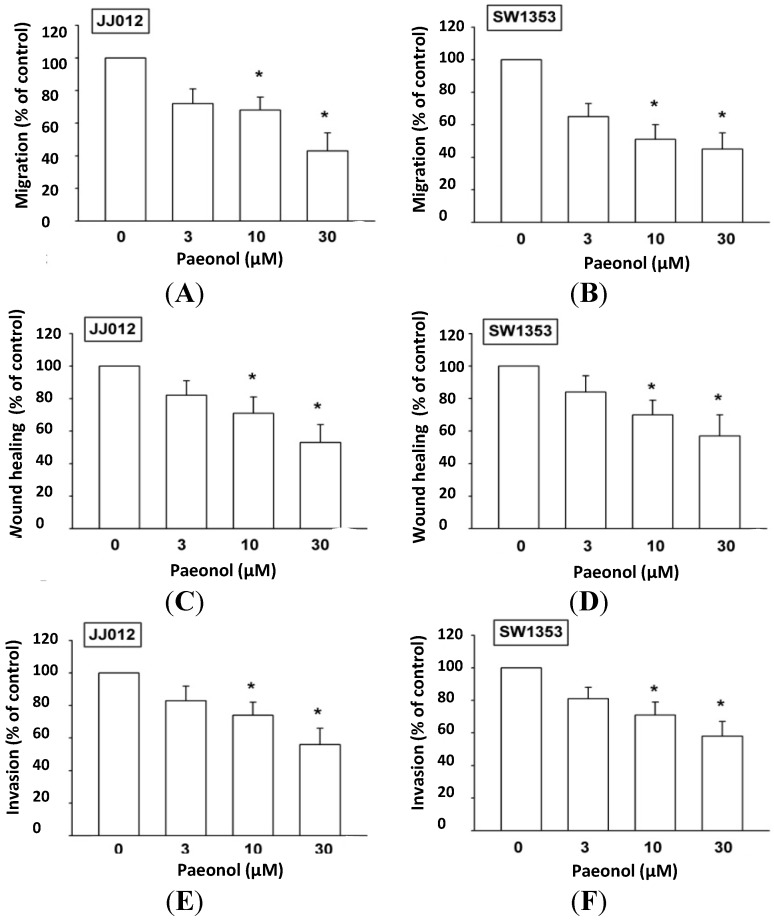
Paeonol inhibits migration and invasion of human chondrosarcoma. (**A**–**F**) Cells were incubated with various concentrations of paeonol for 24 h; cell migration and invasion was examined through Transwell (*n* = 5), wound healing (*n* = 4), and invasion assays (*n* = 5). Results are expressed as the mean ± S.E.M. *, *p* < 0.05 compared with control.

Paeonol has been reported to reduce metastasis of human cancer cells [21,22]. We examined whether paeonol inhibits motility of chondrosarcoma cells. Incubation with paeonol (3–30 μM) dramatically diminished migration in both chondrosarcoma cell lines (Figure 2A,B). In addition, wound-scratching assay confirmed that paeonol reduced wound healing activity in chondrosarcoma cells (Figure 2C,D). We also found paeonol reducing invasive ability via Matrigel basement membrane matrix (Figure 2E,F). Results indicated that paeonol reduces cell migration and invasion of human chondrosarcoma cells.

### 2.3. Paeonol Reduces Motility in Chondrosarcoma Cells by Up-Regulation of miR-141 Expression

**Figure 3:**
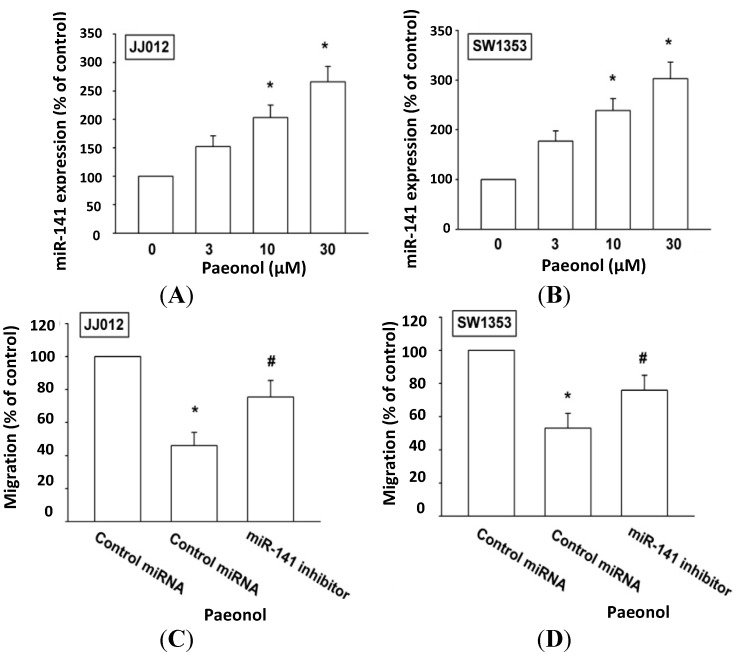
Paeonol increases miR-141 expression in chondrosarcoma cells. (**A**,**B**) Cells were incubated with various concentrations of paeonol for 24 h, miR-141 expression assessed by qPCR (*n* = 6); (**C**,**D**) Cells were transfected with miR-141 inhibitor for 24 h, followed by stimulation with paeonol for 24 h, cell migration examined by Transwell (*n* = 5). Results are expressed as mean ± S.E.M. *, *p* < 0.05 compared with control. #, *p* < 0.05 compared with paeonol-treated group.

The miRNAs have been reported as important regulators of cancer progression and metastasis [23], with miR-141 suggested to inhibit tumor migration and metastasis [24,25]. Up-regulation of miR-141 is thus a novel strategy to suppress tumor motility. We tested paeonol’s reduction of chondrosarcoma metastasis by modulating miR-141 expression to find incubation of chondrosarcoma cells with paeonol raising miR-141 expression in a concentration-dependent manner (Figure 3A,B). To affirm miR-141 involvement in paeonol-inhibited cell motility, miR-141 inhibitor transfection of cells reversed paeonol-suppressed cancer migration (Figure 3C,D). Data suggest paeonol reducing metastasis by up-regulating miR-141 expression.

### 2.4. Paeonol Reduces Activity of Protein Kinase C (PKC)δ and c-Src Signaling Pathways

**Figure 4:**
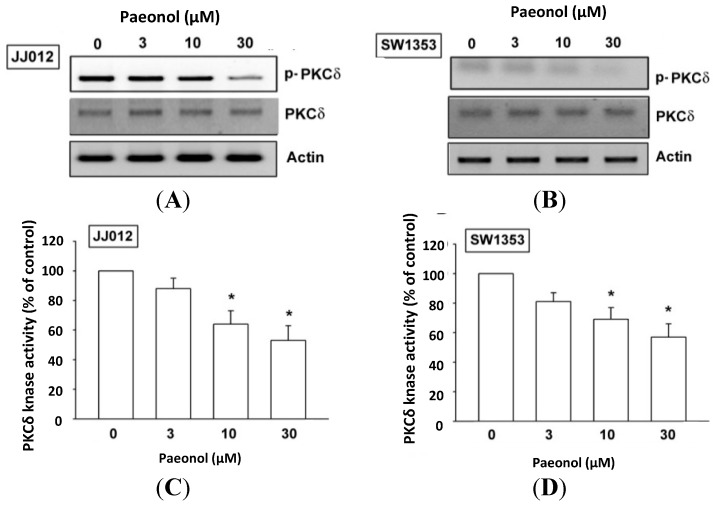
Protein kinase C (PKC)δ involved in paeonol response of human chondrosarcoma. Cells were incubated with various concentrations of paeonol for 24 h; PKCδ phosphorylation and kinase activity was examined by western blot (*n* = 4) and PKCδ kinase activity kit (*n* = 6). Results are expressed as mean ± S.E.M. *, *p* < 0.05 compared with control.

**Figure 5:**
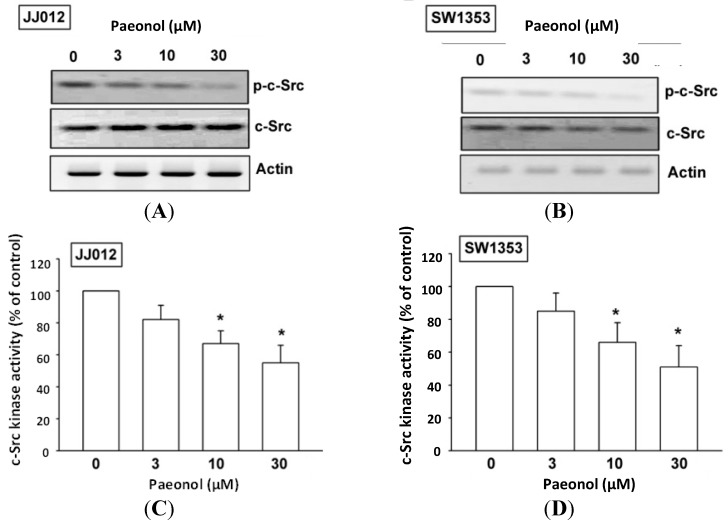
c-Src signaling pathway involved in paeonol response of chondrosarcoma. Cells were incubated with various paeonol concentrations for 24 h, c-Src kinase activity and phosphorylation examined by western blot (*n* = 5) and c-Src kinase activity kit (*n* = 5). Results are expressed as mean ± S.E.M. *, *p* < 0.05 compared with control.

PKCδ-dependent c-Src activation has been reported to mediate metastasis of human chondrosarcoma [26]. After the inhibitory effect of paeonol on cell migration was revealed, its effects on expression of the PKCδ and c-Src pathways were investigated. Incubation of such cells with paeonol significantly decreased PKCδ phosphorylation (Figure 4A,B), while paeonol suppressed PKCδ kinase activity (Figure 4C,D). Likewise, c-Src phosphorylation and kinase activity were abolished by paeonol treatment in a concentration-dependent manner (Figure 5). Paeonol seems to act through a signaling pathway involving PKCδ and c-Src to inhibit migration of chondrosarcoma cells.

## 3. Discussion

Chondrosarcoma, rare but deadly, accounts for nearly 26% of all bone cancers (the second most common type) [27]. Unlike other mesenchymal malignancies, such as osteosarcoma and Ewing’s sarcoma, whose long-term survival dramatically increased with the advent of systemic chemotherapy, data indicate that chondrosarcoma shows a predilection for metastasis to lungs, meaning poor prognosis due to lack of effective adjuvant therapy [28,29]. It is thus crucial to develop effective adjuvant therapy to prevent chondrosarcoma metastasis. Paeonol has various biological activities, such as anti-aggregatory, antioxidant, anxiolytic-like and anti-inflammatory functions [17,30]; it reportedly inhibits tumor formation [20]. Increasing concentration of paeonol (50 μM) slightly suppressed cell viability (~30%) in chondrosarcoma cells (data not shown), indicating that paeonol is not an antitumor agent for chondrosarcoma. Furthermore, paeonol does not have cytotoxic effects in normal chondrocytes and osteoblasts cells (3–30 μM; data not shown). Paeonol has been described to inhibit metastatic potential of human cancer [21,22]. Yet anti-metastasic effects of paeonol on chondrosarcoma cells are mostly unknown. To simply look at the anti-metastasic effects of paeonol, we found that, at non-cytotoxic concentrations (3–30 μM), paeonol reduced human chondrosarcoma cell motility. We found up-regulation of miR-141 through PKCδ and c-Src pathways involved in paeonol-reduced cancer migration. This study identified paeonol as a potential lead base, with good pharmacological properties, on anti-metastasic activity in human chondrosarcoma cells.

Newly identified, small noncoding miRNAs belong to a novel class of regulators that control gene expression by binding to complementary sequences in 3'UTRs of target mRNAs [31,32]. The miR-141 family is well known to inhibit migration and metastasis of human cancer [24,25,33], miR-141 as a negative regulator thereof. We hypothesized miR-141 mediating paeonol-inhibited cancer migration and found that paeonol increased miR-141 expression; transfection with miR-141 inhibitor rescued paeonol-reduced chondrosarcoma metastasis. Results indicate that paeonol reduces chondrosarcoma metastasis through up-regulation of miR-141 expression. miR-141 has been indicated to suppress the migration and invasion of hepatocellular carcinoma cells (HCC) by targeting Tiam1 [24]. Down-regulation of transmembrane-4-L-six-family-1 (TM4SF1) is involved in mR-141-inhibited pancreatic cancer cell invasion and migration [25]. In gastric cancer cells, miR-141 reduced cell motility by targeting hepatoma-derived growth factor (HDGF) [33]. Whether paeonol inhibited chondrosarcoma metastasis by up-regulating miR-141 through targeting these common molecules needs further examination.

PKCδ is proven to mediate tumor migration and metastasis [34], while c-Src (PKCδ-dependent) is involved in cancer migration and metastasis [31,35]. We found paeonol reducing PKCδ phosphorylation and kinase activity in a dose-dependent manner, plus inhibiting c-Src phosphorylation and kinase activity in chondrosarcoma cells. Taken together, our results provide evidence that paeonol down-regulates cell motility in human chondrosarcoma cells via the PKCδ/c-Src signaling pathway. In addition to cancer metastasis, a similar signal pathway has also been reported in the berberine reduced integrin expression and cell migration in chondrosarcoma cells, which inhibited the PKCδ and c-Src pathways [26], prostaglandin E2 regulated oral cancer migration, which involved PKCδ-dependent c-Src pathway [36], and COX-2 promoted cancer metastasis, which mediated PKCδ and c-Src pathways [37]. Taken together, these results show that PKCδ-dependent c-Src activation may have a role in treatment of cancer metastasis.

## 4. Experimental Section

### 4.1. Materials

Paeonol was purchased from Wako Chemicals (Osaka, Japan); rabbit polyclonal antibodies specific for p-PKCδ, PKCδ, p-c-Src, and c-Src from Biotechnology (Santa Cruz, CA, USA); miR-141 inhibitor and *Lipofectamine 2000* from Life Technologies (Carlsbad, CA, USA); all other chemicals from Sigma-Aldrich (St. Louis, MO, USA).

### 4.2. Cell Culture

Human chondrosarcoma cell line JJ012 was kindly provided by the laboratory of Sean P. Scully (University of Miami School of Medicine, Miami, FL, USA) [14], cells cultured in Dulbecco’s Modified Eagle’s Medium (DMEM)/α-MEM supplemented with 10% fetal bovine serum (FBS). Human chondrosarcoma cell line SW1353 was obtained from the American Type Culture Collection; these were cultured in DMEM supplemented with 10% FBS. All cells were maintained at 37 °C in humidified 5% CO_2_ atmosphere.

### 4.3. 3-(4,5-Dimethylthiazol-2-yl)-2,5-diphenyltetrazolium Bromide (MTT) Assay

Viability was rated with a MTT assay. Once treated with paeonol for 48 h, cultures were washed with phosphate-buffered saline (PBS). Then, MTT (0.5 mg/mL) was added to each well, and the mixture was incubated at 37 °C for 2 h. To dissolve formazan crystals, culture medium was replaced with equal volume of DMSO. After shaking the mixture at room temperature for 10 min, absorbance of each well was calculated at 550 nm by microplate reader (Bio-Tek, Winooski, VT, USA) [38].

### 4.4. Triphosphate Nick-End Labeling (TUNEL) Assay

Apoptosis was examined via terminal deoxynucleotidyl transferase-mediated deoxy-uridine TUNEL assay, using BD ApoAlert™ DNA Fragmentation Kit (BD Biosciences, Palo Alto, CA, USA). Cells incubated with paeonol for 48 h were trypsinized, fixed with 4% paraformaldehyde, and then permeabilized with 0.1% Triton-X-100 in 0.1% sodium citrate. After washing, cells were incubated with reaction mixture for 60 min at 37 °C, and stained cells were analyzed by flow cytometer.

### 4.5. Caspase 3 Activity Assay

This is based on the ability of active enzymes to cleave a chromophore from enzyme substrate Ac-DEVD-pNA. Lysates were prepared and incubated with anti-caspase 3. Immunocomplexes were incubated with peptide substrate in assay buffer (100 mM NaCl, 50 mM 4-(2-hydroxyethyl)-1-piperazine-ethanesulphonic acid (HEPES), 10 mM dithiothreitol, 1 mM EDTA, 10% glycerol, 0.1% 3-[(3-cholamidopropyl)-dimethylammonio]-1-propanesulfonate] (CHAPS), pH 7.4) for 2 h at 37 °C. Release of *p*-nitroaniline was monitored at 405 nm; results signify percent change in activity compared to untreated control [39].

### 4.6. Migration and Invasion Assay

Migration assay used Transwell inserts (Costar, NY, USA; 8-mm pore size) in 24-well dishes. For invasion assay, filters were precoated with 30 µL Matrigel basement membrane matrix (BD Biosciences, Bedford, MA, USA) for 30 min. The following procedures were the same for both migration and invasion assays. After treatment with paeonol (3, 10, or 30 μM) for 24 h, cells were harvested and seeded to Transwell at 1 × 10^4^ cells/well in serum-free medium, and then incubated for 24 h at 37 °C in 5% CO_2_. Cells were fixed in 3.7% formaldehyde for 5 min and stained with 0.05% crystal violet in PBS for 15 min. Cells on the upper side of filters were removed with cotton-tipped swabs, filters washed with PBS. Cells on the underside of filters were examined and counted under a microscope. Each experiment (performed in triplicate) was repeated at least three times.

### 4.7. Wound-Healing Migration Assay

For wound-healing migration assay, cells were seeded on 12-well plates at a density of 1 × 10^5^ cells/well in culture medium; 24 h after seeding, the confluent monolayer of culture was scratched with a fine pipette tip. Migration was visualized by microscope, the rate of wound closure observed at the time indicated.

### 4.8. Quantitative Real-Time PCR (qRT-PCR) of mRNA and miRNA

Total RNA was extracted from chondrosarcoma cells by a TRIzol kit (MDBio, Taipei, Taiwan). Reverse transcription proceeded with 1 μg of total RNA and oligo (dT) primer [36,40]. Quantitative real-time PCR (qRT-PCR) analysis used Taqman^®^ one-step PCR Master Mix (Applied Biosystems, Foster City, CA, USA); 100 ng of total cDNA was added per 25 μL reaction with sequence-specific primers and Taqman^®^ probes. Sequences for target gene primers and probes were purchased commercially (GAPDH as internal control) (Applied Biosystems, Foster City, CA, USA), qPCR assays carried out in triplicate by StepOnePlus sequence detection system (Applied Biosystems, Foster City, CA, USA). Cycling conditions consisted of 10-min polymerase activation at 95 °C followed by 40 cycles at 95 °C for 15 s and 60 °C for 60 s. Threshold was set above non-template control background and within the linear phase of target gene amplification to calculate cycle number at which the transcript was detected (denoted *C*_t_) [12].

For miRNA assay, cDNA was synthesized from total RNA (100 ng) by TaqMan MicroRNA Reverse Transcription Kit (Applied Biosystems, Foster City, CA, USA); reactions were incubated first at 16 °C for 30 min, then at 42 °C for 30 min, followed by inactivation at 85 °C for 5 min. Reactions were incubated in a 96-well plate at 50 °C for 2 min, 95 °C for 10 min, followed by 30 cycles of 95 °C for 15 s and 60 °C for 1 min by StepOnePlus sequence detection system. Relative quantification of gene expression was performed with endogenous control gene (*U6*), threshold cycle (*C*_t_) defined as fractional cycle number at which fluorescence passed the fixed threshold. Relative expression was calculated by comparative *C*_t_ method.

### 4.9. Western Blot Analysis

Cellular lysates were prepared, proteins resolved by SDS-PAGE [16,40] and transferred to Immobilon polyvinylidene fluoride membranes. Blots were blocked with 4% bovine serum albumin for 1 h at room temperature, and then probed with rabbit anti-human antibodies against p-PKCδ, PKCδ, p-c-Src or c-Src (1:1000) for 1 h at room temperature (Santa Cruz Biotechnology; Santa Cruz, CA, USA). After three washes, blots were incubated with peroxidase-conjugated donkey anti-rabbit secondary antibody (1:1000) for 1 h at room temperature and visualized with enhanced chemiluminescence, using X-OMAT LS film (Eastman Kodak, Rochester, NY, USA).

### 4.10. Kinase Activity Assay

PKCδ and c-Src activity were gauged by PKC Kinase Activity Assay Kit (Assay Designs, Inc., Ann Arbor, MI, USA) and c-Src Kinase Activity Assay Kit (Abnova, Corp., Taipei, Taiwan). These kits are based on solid-phase ELISA using specific synthetic peptide as substrate for PKCδ or c-Src, and a polyclonal antibody that recognizes the phosphorylated form of substrate.

### 4.11. Transfection with miRNA Inhibitor

Chondrosarcoma cells were transfected with 100 nM of miRNA inhibitor or control miRNA in serum- and antibiotic-free DMEM medium for 4 h using the Lipofectamine 2000 reagent 2 µL, then the transfected cells were allowed to rest for at least 24 h. All stimulations were performed 24 h after transfection.

### 4.12. Statistical Analysis

Data are presented as mean ± S.E.M. statistical analysis of both samples used a Student’s *t* test. Statistical comparisons of more than two groups were performed by one-way analysis of variance with Bonferroni’s *post-hoc* test, and *p* < 0.05 was considered significant.

## 5. Conclusions

It has been recommended that drugs made from natural products play a dominant role in pharmaceutical care. Natural products are vital sources of potential agents for chemotherapy and metastasis [41,42]. This study showed paeonol inhibiting migration and invasion by human chondrosarcoma cells, as well as up-regulation of miR-141 through PKCδ and c-Src pathways involved in paeonol-mediated effects. Evidence indicates paeonol as beneficial in reducing metastasis of human chondrosarcoma.
